# The Functional Microarchitecture of the Mouse Barrel Cortex

**DOI:** 10.1371/journal.pbio.0050189

**Published:** 2007-07-10

**Authors:** Takashi R Sato, Noah W Gray, Zachary F Mainen, Karel Svoboda

**Affiliations:** 1 Howard Hughes Medical Institute, Janelia Farm Research Campus, Ashburn, Virginia, United States of America; 2 Cold Spring Harbor Laboratory, Cold Spring Harbor, New York, United States of America; University of California San Diego, United States of America

## Abstract

Cortical maps, consisting of orderly arrangements of functional columns, are a hallmark of the organization of the cerebral cortex. However, the microorganization of cortical maps at the level of single neurons is not known, mainly because of the limitations of available mapping techniques. Here, we used bulk loading of Ca^2+^ indicators combined with two-photon microscopy to image the activity of multiple single neurons in layer (L) 2/3 of the mouse barrel cortex in vivo. We developed methods that reliably detect single action potentials in approximately half of the imaged neurons in L2/3. This allowed us to measure the spiking probability following whisker deflection and thus map the whisker selectivity for multiple neurons with known spatial relationships. At the level of neuronal populations, the whisker map varied smoothly across the surface of the cortex, within and between the barrels. However, the whisker selectivity of individual neurons recorded simultaneously differed greatly, even for nearest neighbors. Trial-to-trial correlations between pairs of neurons were high over distances spanning multiple cortical columns. Our data suggest that the response properties of individual neurons are shaped by highly specific subcolumnar circuits and the momentary intrinsic state of the neocortex.

## Author Summary

Mice depend on their whiskers to explore their environment. Tactile receptors at the base of each whisker relay sensory information to a brain area called the barrel cortex. This somatosensory area consists of an orderly array of cortical columns, each containing clusters of neurons whose responses are driven primarily by stimulation of a particular whisker, in addition to stimulation of surrounding whiskers. The detailed structure of this cortical map, especially within a column, is poorly understood. We imaged multiple neurons loaded with calcium indicators to monitor whisker deflection-evoked action potentials in the barrel cortex of mice. Calcium imaging methods allowed us to reliably detect action potentials in approximately half of the cortical neurons. For these neurons, we measured the spiking probability following whisker deflection and thus created a high-resolution map of whisker selectivity. On average, the whisker map varied smoothly across the surface of the cortex. But the whisker selectivity of individual neurons differed significantly, even for neighboring neurons. The responses of neurons, even those that were distant from each other, were highly correlated across trials and depended on the level of overall brain activity at the time of the stimulus. Our data suggest that the response patterns of cortical neurons are determined by specific local circuits and by the global state of the cortex, which changes over time.

## Abstract 3

In vivo two-photon calcium imaging in layer 2/3 of the mouse barrel cortex uncovers highly heterogeneous receptive field properties of neighboring neurons in response to whisker stimulation but long-range correlations in neural responses across cortical columns.

## Introduction

In sensory cortical areas, neurons that respond to similar stimuli are clustered together in vertical cortical columns [1–5]. Cortical columns are typically arranged in maps, so that columns with similar response properties are close to each other along the cortical surface [2,6–9].

Most of our knowledge about cortical maps comes from measurements with limited spatial resolution. Single-unit measurements sample neurons over distances of 100 μm or more [10,11]. In addition, blind extracellular recordings are biased towards neurons with strong responses [10,12–14]. Optical imaging of intrinsic signals and voltage-sensitive dyes average the responses over large populations of neurons [6,7,15–18]. We therefore know little about the organization of cortical maps with single-cell resolution. Recently, bulk-loading of Ca^2+^ indicators, in combination with two-photon microscopy [19–22], has been used to analyze the microstructure of visual cortical maps at the level of individual neurons [23,24].

Cortical maps and the underlying circuits have been studied extensively in the barrel cortex of rodents [25], in which neurons in each cortical column are driven best by the column's principal whisker (PW), and more weakly by surrounding whiskers [26,27]. Neurons in layer (L) 4 are clustered in anatomical barrels, each corresponding to a particular PW. Between L4 barrels are narrow septa [25,28]. L4 barrels are the targets of thalamocortical axons from the ventral posterior medial nucleus (VPM, “lemniscal” pathway). Layer 2/3 (L2/3) pyramidal neurons receive strong columnar input from L4, the major ascending projection in the cortex. Neurons in L5A receive strong input from posterior medial nucleus (POm, “paralemniscal” pathway) and project to L2 [29]. L2/3 cells are therefore key components in some of the earliest stages of intracortical processing [26,27,30–32] (for review, see [33]). L2/3 receptive fields are broader than L4 receptive fields. L2/3 neurons also exhibit robust experience-dependent plasticity [34–42].

We used in vivo two-photon Ca^2+^ imaging to examine the microstructure of the barrel cortex map in L2/3 of the mouse. Since L2/3 cells fire few (0–2) action potentials (APs) in response to whisker stimulation [26,27], we developed imaging and analysis methods to detect single APs in individual neurons. Superposed on a coarse map of whisker dominance, we find locally highly heterogeneous response properties.

## Results

### Spontaneous and Whisker Deflection-Evoked Fluorescence Transients

**Figure 1:**
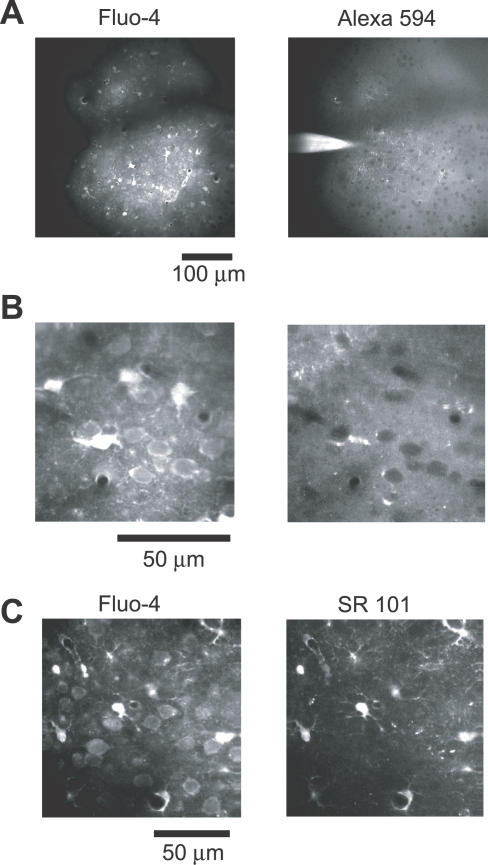
Loading Populations of L2/3 Neurons with Ca Indicators In Vivo (A) Left: cortical region stained with Fluo-4 AM (green fluorescence). Right: the distribution of Alexa 594 during loading (red fluorescence). The dark band is a shadow cast by a blood vessel in the imaging area. (B) Higher magnification of cortical cells. Left: Fluo-4 AM image. Right: Alexa 594 image. Note the clear correspondence between the labeled cells in the Fluo-4 AM image and the unlabeled dark cells in the Alexa 594 image (right). Other dark structures in the Alexa 594 image correspond to blood vessels viewed in cross-section. (C) Cells stained with Fluo-4 AM (left) and Sulforhodamine 101 (SR 101) (right). The cells with high Fluo-4 AM fluorescence were also labeled with Sulforhodamine 101, indicating that they were astrocytes.

To investigate the fine-scale functional organization of the barrel cortex, we imaged AP-evoked [Ca^2+^] transients in populations of L2/3 neurons in the barrel cortex. Cells were loaded with Fluo-4 AM using multicell bolus loading [22]. Fluo-4 AM (green; 1 mM) and Alexa 594 (red; 0.05 mM) were pressure-injected (5 psi, 10 ms, 10–20 pulses) into L2/3 of the somatosensory cortex in anesthetized young mice (postnatal day 18–25). This procedure loaded a cluster (diameter, approximately 200 μm) of neurons with Fluo-4 AM (Figure 1A). Loaded neurons displayed relatively homogeneous, dim green fluorescence against a highly heterogeneous fluorescent background. The background is presumably due to the presence of labeled neuropil, including axons and dendrites [43]. In the red channel, neurons appeared as dark objects against a light background (Figure 1B). The red fluorescence disappeared within 30 min of loading by diffusion.

Astrocytes were distinguished from neurons by their morphology and bright fluorescence in the green channel, possibly due to higher resting [Ca^2+^] [22] or more efficient uptake of the calcium indicator [44]. In some experiments, we confirmed the identity of astrocytes by co-labeling with Sulforhodamine 101, a red fluorescent dye that selectively labels astrocytes (Figure 1C) [45].

**Figure 2:**
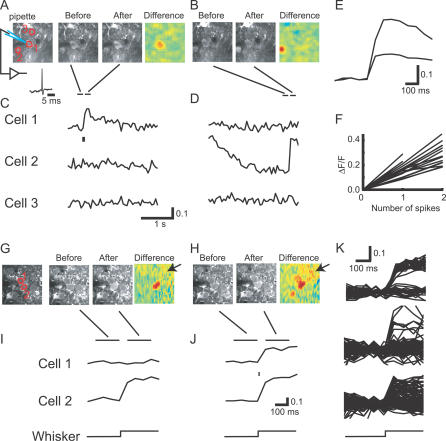
Spontaneous and Whisker Stimulation-Evoked Fluorescence Transients in the Somata of Cortical Neurons (A) Images of spontaneous fluorescence transients. The left image shows three regions of interest corresponding to three cells over 3 s. The electrophysiological recording was performed from Cell 1. The subsequent two images show the fluorescence before and after onset of the transient in Cell 1, averaged over the time periods indicated by horizontal bars in (C) and (D). The third image shows the difference image. (B) Different 3-s trial, same region as in (A). (C) Fluorescence changes corresponding to (A). The tick mark indicates an AP recorded from Cell 1 using a cell-attached electrode. See also Video S1. (D) Fluorescence changes corresponding to (B). In this trial, Cell 1 did not produce an AP. (E) Averaged fluorescence transients following one and two spikes (Cell 1 in [A–D]). The ratio of the amplitudes is a factor of two. (F) The peak fluorescence change as a function of the number of APs (18 cells). (G and H) Examples of whisker stimulation-evoked fluorescence transients. Cell-attached recordings were made from Cell 1. In (G), only Cell 2 produced a fluorescence transient. In (H), both cells produced a transient. Note the diffuse neuropil signal (arrows). (I and J) Fluorescence changes corresponding to (G) and (H), respectively. Whisker stimuli are indicated on the bottom. (K) Fluorescence traces from different trials were overlaid. Top, traces corresponding to Cell 1 (G–J). Trials producing whisker stimulation-evoked transients (successes) were easily distinguished from trials without transients (failures). Middle and bottom, traces from other cells. In these cells, the separation between successes and failures was less clear.

Labeled neurons often displayed spontaneous transient increases in the green fluorescence signal (Figure 2A and 2B; Video S1). These saw-tooth–shaped fluorescence transients had a rapid onset (<64 ms, the sampling interval) and decayed relatively slowly (time constant of approximately 1 s). The time course of these transients suggests that they were caused by APs [22,43,46–49]. APs depolarize the neuronal membrane and briefly open voltage-gated calcium channels to admit Ca^2+^. The decay time constant of the transients likely reflects clearance of Ca^2+^ from the neuronal cytoplasm [50,51].

**Figure 3:**
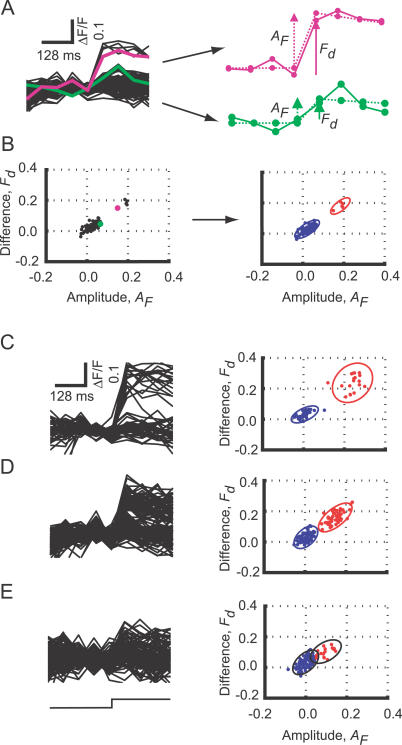
Distinguishing Successes and Failures in Response to Whisker Deflection (A) Left: multiple fluorescence transients from a single cell aligned on the whisker stimulus. Right: analysis of two representative trials (green and pink traces on the left). For each trace, two values, the difference *F*_d_ (solid arrows) and the amplitude *A*_F_ (dashed arrows) were computed. The dotted lines are the results of template matching. (B) Left: the *F*_d_ and *A*_F_ were plotted for each trial. Right: these points were clustered into two groups (red, successes; blue, failures). The 95% confidence ellipses are overlaid on the graph. The green and pink dots are from the two representative trials shown as the green and pink traces in (A), respectively. (C–E) Analysis for three different cells. (C) Well-separated cell. (D) Marginally separated cell. (E) Example of a cell in which successes and failures overlapped.

**Figure 4:**
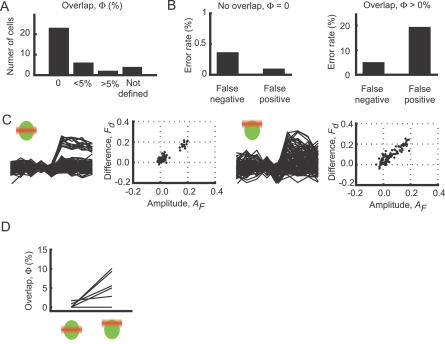
Validation of the Analysis Algorithms with Cell-Attached Recordings (A) The distribution of the overlap between the two ellipses for experiments with cell-attached recordings (*n* = 34 neurons). For some cells, the number of APs was too little to allow analysis (not defined). (B) For cells with zero overlap, the correspondence between scoring successes and failures from imaging experiments and APs was very high (left). For cells with overlap, false-positive and false-negative trials were often detected (graph on right). Note the differences in *y*-axes between the two graphs. (C) The location of the focal plane within the soma affects detection fidelity. When the focal plane was centered on the soma (left), successes and failures were clearly distinguishable. In the same cell, when the focal plane was closer to the upper edge of the cell (right), the two clusters were less segregated. (D) The overlap increases, and detection fidelity decreases, as the focal plane is moved from the center of the soma, closer to the edge.

To characterize the relationship between fluorescence transients and neuronal activity, we recorded spikes from labeled neurons in loose-seal cell-attached mode under visual control (Figures 2–4, see Materials and Methods). Comparisons of spike trains and fluorescence signals from the same cell revealed that they were highly correlated (Figure 2C and 2D, Cell 1). Clear fluorescence transients were only seen after APs. The amplitudes of fluorescence transients following spike doublets were larger than the fluorescence signals following single spikes (Figure 2E) (singlet, 15.7 ± 5.0%, *n* = 19 cells; doublet, 28.6 ± 8.0%, *n* = 16 cells) (Figure 2F). Therefore, cytoplasmic fluorescence transients report spiking activity [43,52]

We next examined the fluorescence transients evoked by single-whisker deflections. Electrophysiological studies have revealed that barrel cortex neurons respond with one, or at most two, APs a short time (10–30 ms) after whisker stimulation [27,30]. More-recent studies indicate that neurons often fail to respond to individual stimulus trials, and some neurons do not respond to whisker stimulation at all [12,32]. In our experimental condition, using cell-attached recording, we confirmed that out of 2,424 trials (34 neurons), almost all success trials (873/885, 99.6%) corresponded to a single AP. The spike latency was 24 ± 6 ms [32]. Consistently, labeled neurons exhibited sensory stimulation-evoked fluorescence transients in some trials (successes), but not in others (failures) (Figure 2G–2J). Overlaying all (50–300) trials from individual cells revealed that successes and failures can be easily distinguished in some neurons (Figure 2K, top). In other neurons, successes and failures overlapped (Figure 2K, bottom), indicating that successes may be difficult to separate from failures in these cells.

### Decoding Whisker Deflection-Evoked Fluorescence Transients

We developed algorithms to identify the neurons in which individual APs can be reliably detected based on fluorescence measurements, and to separate successes and failures in these cells. Each trial (duration approximately 0.5 s) consisted of four prestimulus images followed by four poststimulus images (64 ms per image). From each trial, we extracted two parameters (Figure 3A):

First, the change in Δ*F/F* between the last prestimulus frame and the first poststimulus frame was calculated. Since spikes can occur up to about 50 ms after whisker deflection [32,53], we also computed the difference between the first and second poststimulus frames. *F*_d_ was defined as the larger of these values.

Second, the amplitude, *A*_F_, of the fluorescence transient was derived from template matching [54]. The template was as follows:

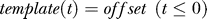


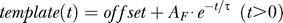



The time constant of the template, τ, was the decay time of the exponential fluorescence transients (1 s). The origin (0 ms) of the template window (−192 ms to 256 ms, eight frames) was positioned at the frame of interest, and *A*_F_ and *offset* were adjusted to fit the data by minimizing the sum of squared errors.

We plotted *F*_d_ and *A*_F_ for all trials (Figure 3B) and applied hierarchical clustering, using the Euclidean distance to define the distance between points. For many cells, the trials fell into two groups, corresponding to failures and successes. To quantify the separation between these groups, we calculated the 95% confidence ellipse for each of the two clusters (Figure 3B). We computed the overlap of the ellipses, Φ, as a measure of the fidelity of separating failures and successes. In a fraction of cells, the two confidence ellipses were separated (Φ = 0), indicating that successes and failures could be identified with few errors (Figure 3C and 3D). In other cells, the ellipses overlapped (Φ > 0), indicating that successes could not be separated from failures with high confidence (Figure 3E).

To test the performance of our imaging method and analysis algorithm, we compared spike detection based on Ca^2+^ imaging with loose-seal cell-attached recordings from the same cells (*n* = 34). For imaging, the focal plane was positioned at the equator (center) of the soma of the recorded cell. In 68% of these cells, the two confidence ellipses were nonoverlapping (*n* = 23) (Figure 4A), and our algorithm produced negligible error rates in this subpopulation (2/546 trials were false negative; 1/1,105 trials was false positive) (Figure 4B, left). For the other 32% of cells with partially overlapping confidence ellipses, the error rates were higher (24/469 trials were false negative; 170/873 trials were false positive) (Figure 4B, right). This analysis shows that successes and failures can be accurately separated in two-thirds of randomly selected neurons. Furthermore, neurons in which successes and failures are separable can be unambiguously identified using a simple analysis of *F*_d_ versus *A*_F_ plots.

We next examined the factors limiting reliable spike detection in a subset of cells. We noticed that in the *F*_d_ versus *A*_F_ plots, the points corresponding to failures were not uniformly distributed around zero; even during failures, the fluorescence signal tended to increase after whisker deflection (Figure 3B–3E). *F*_d_ and *A*_F_ were significantly larger than zero for 32 and 24 of the 34 recorded neurons, respectively (*p* < 0.05, *t*-test). For several reasons, we believe this noise is due to the “neuropil signal” [43]. When imaging deep in tissue, the two-photon excitation volume is spatially extended; along the beam direction, its size can be on the order of 4–5 μm (unpublished data). The excitation volume therefore partially overlaps with neuropil outside of the targeted somata (Figure 4C and 4D). Since the neuropil consists of labeled axons and dendrites, which can produce large AP-evoked [Ca^2+^] changes [55,56], it shows physiological [Ca^2+^] changes (Figure 2G and 2H, arrows). The performance of our spike detection algorithms should therefore depend on the relative positions of the focal plane and the target soma: in cases in which the focal plane overlaps the equator of the cell, most of the excitation volume will be inside the soma, and the neuropil signal will contribute minimally, implying optimal spike detection; when the focal plane is closer to the edge of the cell, the neuropil signal will contribute more signal, which in turn will lead to a degradation of spike detection. We tested this hypothesis directly by measuring the overlap between the failure and success ellipses while changing the focal plane. The separation between failures and responses decreased when the focal plane was moved from the equator towards the edge of the cell by 5 μm (*n* = 7, *t*-test, *t*_6_ = 2.67, *p* < 0.05) (Figure 4C and 4D).

In addition to the neuropil signal, other factors limit the separation of successes and failures. The amplitudes of the fluorescence changes following APs differ from cell to cell (Figure 2F). Even among the cells that exhibited clear segregations, *A*_F_ varied from neuron to neuron (10th percentile, 15.5%; 90th percentile, 25.9%; mean 20.8%), and cells with smaller amplitudes showed less separation. We note that in experiments in which cell-attached recordings and imaging were combined, the focal plane was aligned with the center of the neurons (Figure 4A and 4B). Under typical conditions for multicell imaging, the focal plane overlaps in a random manner with imaged neurons, and the fraction of neurons in which successes and failures can be accurately separated is expected to be somewhat less than two-thirds. For the rest of the study, we focused on the neurons in which failures and successes could be clearly distinguished.

### Microorganization of the Barrel Cortex

**Figure 5:**
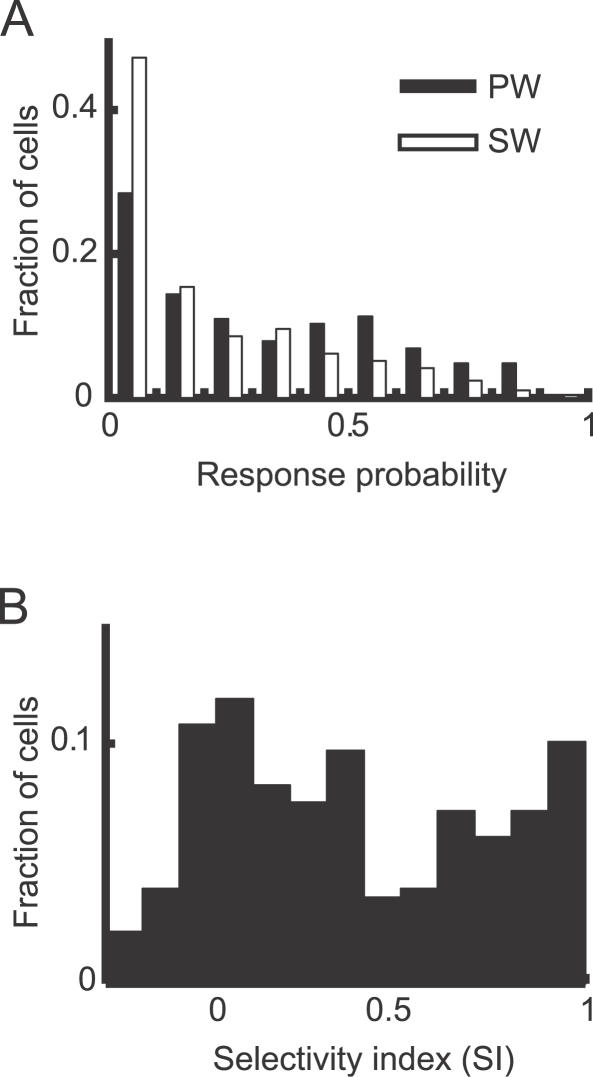
The Response Properties of L2/3 Neurons (A) The distribution of success probabilities in response to PW and SW stimulation (*n* = 292 neurons). (B) The distribution of the SI, defined as the relative response probability for the PW and SW (SI: possible range, −1,1).

We examined the spatial organization of the response properties of L2/3 neurons in the barrel cortex. Using local field potential (LFP) measurements, we determined the PW and the surround whisker (SW) evoking the largest response. Out of 668 neurons we imaged, 292 neurons (43.7%) exhibited separable successes and failures (i.e., nonoverlapping ellipses in the cluster analysis of *F*_d_ versus *A*_F_ plots, Figure 3). On average, the response probability (*P*_r_) was 0.32 ± 0.26 (*n* = 292 cells) for PW stimulation, and 0.20 ± 0.22 (*n* = 292 cells) for SW stimulation. We calculated the selectivity index (SI), which represents the relative response probability for the two whiskers (see Materials and Methods). As expected, the majority of cells were dominated by the PW (SI > 0). However, *P*_r_ and SI varied greatly from cell to cell (Figure 5A and 5B). For the analysis of SI, we will focus on the neurons in which, in addition to the separability criterion, at least 20 successes were detected (191 out of 292 neurons).

What could be the sources of variability in whisker dominance? The SI is known to depend on the position of the neuron within the barrel [30]. It is possible that somatotopy varies smoothly within a barrel column: neurons nearer to the border with the barrel column dominated by the SW would respond relatively more to the SW, at the expense of the PW, and thus have lower SI values.

**Figure 6:**
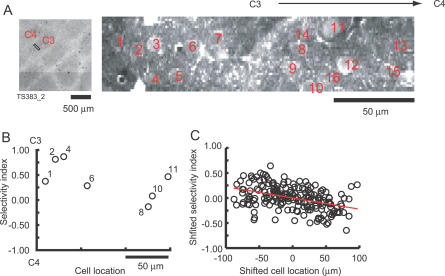
Somatotopy at the Level of Single Cells (A) Left: the location of the imaged area in CO-stained barrels. Right: Fluo-4 AM image showing the locations of the analyzed cells. The imaged area was centered on the C3 barrel, with the C4 barrel to the right. (B) The SI of neurons in (A) as a function of their location. Only cells without overlap (Φ = 0) were included in the analysis. (C) Spatial changes in the selectivity index across 33 experiments. To overlay different experiments the SI and location were shifted so that the center of gravity of the data points was on the origin.

We measured SI as a function of distance across whisker rows (rostral–caudal). In the example of Figure 6, we imaged 16 neurons in the C3 barrel. The largest response was evoked by the C3 whisker; the C4 barrel is to the right of the C3 whisker (Figure 6A). Seven out of 16 neurons responded to whisker stimulation and produced signals that allowed us to separate failures and successes. The SI varied greatly from cell to cell, even for adjacent cells (Figure 6B). We combined the data from all experiments (*n* = 33, 191 neurons), using the center of mass of the data points as the reference point or origin. This analysis showed that SI changed with distance across a barrel (*p* < 0.001) (Figure 6C). The spatial gradient in SI, −0.22/100 μm, is consistent with the spacing between barrel columns (~300 μm) and the average SI within the barrel (0.28).

Recent anatomical studies have shown that L2 and L3 cells in the mouse barrel cortex are part of distinct thalamocortical circuits [29]. L3 cells are part of the “lemniscal” pathway: they are dominated by L4, which receives direct input from VPM. L2 cells are excited by lemniscal L3 cells and by L5A cells which, like L3 cells, are excited by L4 cells. However, L5A cells also received strong direct input from the posterior medial nucleus (POm), which is part of the paralemniscal circuit. L2 cells therefore have a mixed character (lemniscal and paralemniscal). The thalamocortical projection originating in POm is broad [29], suggesting that if L2 cells are primarily driven by POm, we would expect smaller SI gradients in L2 than L3. However, the spatial gradient in SI was indistinguishable for L2 and L3 cells (L3, >160 μm from the pia, *r* = −2.59/mm, *n* = 14, 79 cells; L2, <130 μm from the pia, *r* = −2.16/mm, *n* = 10, 64 cells; *p* > 0.1, bootstrap analysis). These findings suggest that both L3 and L2 are functionally primarily lemnsical under our experimental conditions.

We next examined whether there is a clear functional border in L2/3 corresponding to the anatomical border between barrels in L4. We mapped the locations of imaged areas (*n* = 27, 160 neurons) and divided the cells into those that were located within 60 μm of the barrel border and those that were located more than 100 μm from the border, close to the barrel center. We did not find a difference in the gradient in SI between these two populations (*p* > 0.05, bootstrap analysis). Therefore, somatotopy varies relatively smoothly across the borders between barrel columns.

**Figure 7:**
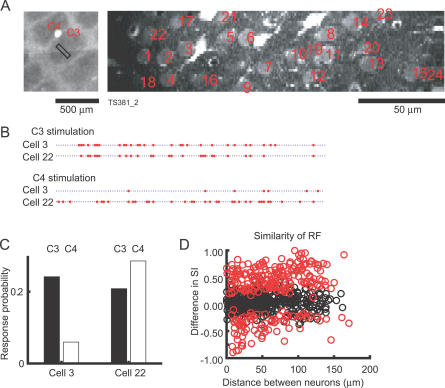
Heterogeneity of Response Selectivity (A) Left: the location of the imaged area in CO-stained barrels. Right: Fluo-4 AM image showing the locations of the analyzed cells. (B) The response pattern of two neighboring neurons (Cell 3 and Cell 22) to stimulation of whiskers C3 (the PW) and C4. (C) The response probability of Cell 3 and Cell 22 to C3 stimulation (black) and C4 stimulation (white). (D) The difference in SI for pairs of neurons as a function of the distance between the neurons. The distance was calculated as the projection of the position vector connecting pairs of neurons onto the line that connects the two neighboring barrels. Essentially indistinguishable data were obtained using the absolute distance (length of the position vector; see Figure S3). Red circles indicate pairs of neurons whose SIs were significantly different. RF, receptive field.

Changes in somatotopy accounted for only a small fraction of the heterogeneity in responses to whisker deflection. Even neurons in close proximity (less than 30 μm) frequently exhibited substantially different receptive field structure (Figure 7A–7C). We compared the SIs of a large number of neuronal pairs as a function of distance between the neurons. We found that 264/654 (40%) pairs had significantly different SIs (*p* < 0.05, bootstrap). When we restricted our analysis to pairs of neurons located within 50 μm of one another, the SIs were still significantly different for many neuronal pairs (112/321, 35%). This indicates that the response properties of nearby neurons are highly heterogeneous, even if they are intermingled in the same cortical column.

### Correlated Activity in the Barrel Cortex

**Figure 8:**
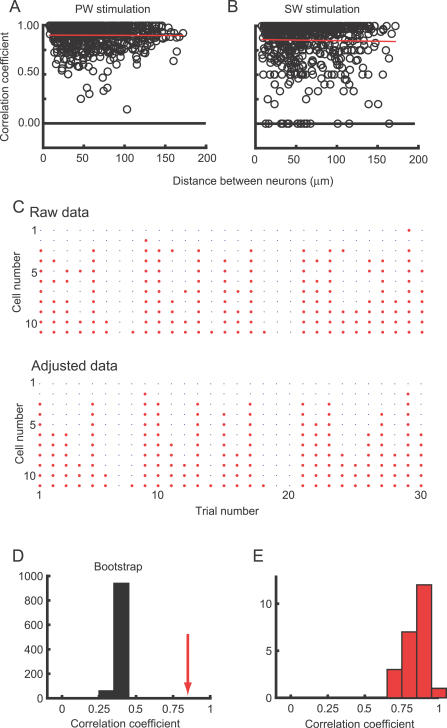
Trial-to-Trial Correlation of Sensory-Evoked Responses (A) The trial-to-trial correlation between the response patterns for each pair of neurons (imaged simultaneously) to PW stimulation was plotted as a function of the distance between the two neurons. (B) The same analysis as depicted in (A), but with the paired correlations plotted from the response pattern following SW stimulation. (C) Top: raw response pattern of 11 simultaneously imaged neurons to PW stimulation. Each row represents one neuron, and each column represents one trial. The neurons were sorted based on response probability (Cell 1 responded the least, and Cell 11 responded the most). Bottom: the response patterns from above were sorted so that all responsive trials were clustered towards the bottom of the chart, regardless of cellular identity and origin of that response. The number of responsive neurons in each trial remained the same. (D) Monte Carlo simulation for the correlation coefficient between “Raw data” and “Adjusted data,” from (C). The red arrow indicates the actual value. (E) The distribution of the correlation coefficient between “Raw data” and “Adjusted data” for 23 imaging sessions in which more than five neurons responded for more than 20 trials.

We next examined the trial-to-trial variability across neurons. Although neurons exhibited different whisker selectivities, they tended to respond on the same trials (Figure 7B). To quantify the strengths of trial-to-trial correlations, a commonly used method is to calculate the correlation coefficient of two vectors corresponding to each neuron [57,58]. However, the correlation coefficient is determined in part by the differences in the response probabilities of the cells. For example, if one neuron fires frequently and the other rarely, then the correlation coefficient is low, even if they were highly correlated. We thus used a different measure of correlation that corrects for differences in response probabilities. We define the correlation between two neurons simply as the number of trials in which both of the compared neurons fired, divided by the number of trials in which the less-responsive neuron fired. Using this definition, we found that the firing correlation was very high (PW: 0.90 ± 0.12; SW: 0.85 ± 0.22, *t*-test, *n* = 640; *p* < 0.001) (Figure 8A and 8B). In other words, if the less-responsive cell fires in a particular trial, the more-responsive neuron will also fire with high probability. The pairwise correlation did not depend on the distance between the neurons (regression test; *p* > 0.9 for PW, *p* > 0.5 for SW), indicating that all imaged neurons were influenced by state fluctuations in the cortex that have space constants that are much larger than one barrel. The highly correlated activity was also observed under isoflurane anesthesia (0.93 for PW, 49 pairs, four imaging sessions)

We further quantified trial-to-trial fluctuations to test whether the cells that are more responsive likely fire if the less-responsive cells fire. We constructed a response matrix based on the raw data (Figure 8C): In the top panel, neurons are ordered based on their response probability (bottom, highest; top, lowest). It can be seen in this example that in most of the trials in which Cell 3 fired (see Figure 8C, top) (trials 1, 5, 9, 10, 11, 13, 17, 21, 23, 24, and 29), cells 4–11 also fired, with few exceptions. We then rearranged the same dataset by assigning the responses on each trial to the most-responsive neurons (preserving the total number of responses). In other words, we shifted the responses downward in the matrix. This procedure produces a matrix of spiking activity in which the least-active cell always predicts a spike in more-active cells (Figure 8C, bottom). The rearranged response matrix was very similar to the raw response matrix (*r* = 0.85; *p* < 0.01, compared to shuffled data, see Materials and Methods for details; Figure 8D, red arrow). Across 23 experiments in which more than five neurons responded in more than 20 trials, the correlation coefficient was 0.85 ± 0.08 (Figure 8E). Thus, in most cases, whenever a particular neuron fires, there is a very high probability that other neurons with the same or greater overall response properties will also fire.

Previous studies on the barrel cortex have found that the magnitude of the sensory-evoked response depends on whether the cortex is in an UP or DOWN state [59,60]. Consistent with these studies, the correlation coefficient between the number of responsive neurons and the LFP amplitude were correlated (mean *r* = 0.14, *t*_20_ = 25.3, *p* < 0.001), indicating that neurons were more likely to fire when the stimulus was delivered during a DOWN state (Figure S1). Therefore, the variability in the sensory responses is at least in part due to fluctuations in the cortical state.

## Discussion

### Imaging the Dynamics of Multiple Single Neurons at the Level of Single Spikes

In many areas of the cerebral cortex, the AP rates are low (around 1 Hz), and sensory information is encoded by the presence or absence, or the timing, of individual APs [12,32,61]. Reading out the neural code therefore demands the detection of single spikes in multiple individual neurons. Here, we used bulk loading of Ca^2+^ indicators and two-photon microscopy to detect APs in multiple neurons. In about half of the neurons, it was possible to detect single APs, with negligible error rates.

Previous studies have used bulk loading with Fura-2 AM or Oregon Green BAPTA-1 AM. AP-evoked fluorescence changes have been characterized in the rat barrel cortex loaded with Oregon Green BAPTA-1 AM, in which single APs could be detected with more than 95% fidelity [43] Under our experimental conditions, loading with Fluo-4 AM, rather than Oregon Green BAPTA-1 AM, provided improved signal-to-noise ratio and AP-detection efficiency. This is expected since Fluo-4 provides much larger fluorescence changes upon Ca^2+^ binding than Oregon Green BAPTA-1 [62,63]. Nevertheless, in about half of the imaged neurons, we were not able to detect APs reliably without introducing considerable error rates (>0.5%). The difference in the detection efficiency between Kerr et al. [43] and our data could be due to two reasons. First, Kerr et al. reported on cells that were optimally sectioned by the focal plane, whereas we report on all discernable somata. With optimal sectioning, we were able to detect spikes reliably in two-thirds of the recorded cells. Second, species-specific differences likely play a role. Kerr et al. imaged rat neurons, whereas we imaged mouse neurons; for larger neurons, the contributions of the neuropil signal are expected to be further reduced, enhancing the detection efficiency. In addition, the amplitudes of Fluo-4 fluorescence signals in the mouse suggest that the AP-evoked Ca^2+^ accumulations are smaller and more variable across cells than in the rat, compromising AP detection in some cells. Despite these challenges, we have shown that it is possible to use Ca^2+^ imaging to measure spiking in multiple neurons in a small volume of mouse barrel cortex.

We note that our approach for detecting single APs relies on the low firing rates seen in the barrel cortex [32]. Moreover, although our method distinguishes between trials with and without APs, it cannot reliably distinguish the number of APs in a short burst. Therefore, our approach cannot be generally applied to the analysis of spike trains [64].

### Microstructure of Somatosensory Maps

Maps of response selectivity are thought to vary continuously across the cortical surface, with some notable discontinuities, such as fractures and pinwheels [7,65,66]. However, the vast majority of mapping studies have employed techniques that average the activity of neurons distributed over distances of 100 μm or more and thus wash out the microstructure of cortical maps. Bulk loading of Ca^2+^ indicators, in combination with two-photon microscopy [22], has made possible the analysis of the microstructure of cortical maps, at the level of individual neurons [23,24]. Additional technical refinements facilitating single-spike detection have allowed us to analyze the microstructure of the mouse somatosensory cortex.

Single-unit recordings from rat and mouse barrel cortex under various conditions have reported that single-whisker deflections evoke approximately one AP [26,27,30,67]. Recent studies using in vivo whole-cell or loose cell–attached recordings reported much lower levels of activity (0.14 AP per whisker deflection for L4 and 0.03 AP per whisker deflection for L2/3); many cells exhibit only subthreshold responses [12,32]. This discrepancy is most likely due to the sampling biases of single-unit recordings, which are insensitive to nonspiking cells [13,14].

In the current study, the response probability to the PW was 0.32 on average. The difference between our results and previous studies using whole-cell recordings [12,32] is likely due to differences in the anesthesia protocols; in our preparation, the sensory-evoked response is stronger under ketamine–xylazine anesthesia than under urethane or isoflurane anesthesia (unpublished data).

The response probability to the PW and SW varied greatly across neurons. At the level of neuronal populations, receptive fields changed gradually with distance within barrels. This is consistent with previous single-unit studies in the rat that have shown that the SW response depends on the location of the recording electrode within the barrel; at a particular location, the SW dominating the nearest surround column tends to evoke the largest surround response [30]. In addition, receptive fields changed gradually across borders between barrel columns, demarcated by the narrow septa in L4. This is consistent with the functional anatomy in the mouse barrel cortex, in which L3 (L2) cells above septa and barrels are coupled to L4 (L5A) cells in a similar manner [29]. The wiring differs in the rat: L2 and L3 neurons in barrel-related columns receive strong input from L4 barrels [68–72], with a minor input from L5A. In contrast, L2 neurons in septum-related columns receive strong input from regions in L5A below septa [68]. L3 neurons in septum-related columns are only weakly coupled to intracortical circuitry in brain slices [40]. We predict that the response properties in L2/3 of the rat barrel cortex could be qualitatively different above septa and above barrels [32].

The spatial gradients associated with somatotopy only explained a small fraction of the cell-to-cell variability in response selectivity. A key finding from our current study is that neighboring neurons, even if located within 50 μm, can have dramatically different receptive fields. Previous studies using extracellular recordings have reported that pairs of neurons recorded on the same electrode can exhibit different response selectivities [57,58]. However, interpreting the electrophysiological data is challenging since extracellular electrodes sample APs from large volumes of tissue (up to 100 μm or more; [11]). Our findings are analogous to mapping studies in the rodent visual system, where neighboring neurons can have highly distinct orientation selectivity [24,73,74].

What features of cortical circuits could underlie locally heterogeneous response properties? Recently, several studies in brain slices have revealed that cortical columns contain highly specific, fine-scale subnetworks [71,75–77]. For example, L2/3 neurons that are connected with each other are more likely to receive common input from L4 than unconnected neurons [78]. It is likely that the heterogeneous and spatially intermingled response properties that we observe are a reflection of this fine-scale specificity.

### Long-Range Correlations in Sensory Responses

Even the earliest single-unit studies have noted that cortical responses to sensory stimulation vary substantially from trial to trial [79–82]. Response variability is often thought of as noise. A common assumption is that the brain averages over neurons to reduce noise and improve signal detection [57,83]. However, averaging works only if individual neurons respond independently.

We find remarkably high correlations between the responses of individual neurons. Furthermore, the strength of the correlations did not attenuate with distance up to 150 μm between neurons. The highly correlated sensory responses observed in our study suggest that population averaging is unlikely to aid signal detection in the barrel cortex of mice.

Consistent with previous studies [59,60,84–87], we found that the response variability of individual neurons is reflected in the response pattern of the local cortical network. Particularly, in the barrel cortex, it has been reported that the magnitude of the sensory-evoked response is smaller when the cortex is in the UP state compared to when the cortex is in the DOWN state [59,60]. Our findings are consistent with these studies. However, these previous studies were not able to assess whether all neurons, or a subset of neurons, contribute to the correlations. Our dense sampling of neurons revealed that all responding neurons show similar trial-to-trial fluctuations.

Our study, as well as most of the previous work on response variability in population of neurons, was carried out in anesthetized animals. The behavioral significance of our findings will require future studies with awake animals performing sensory tasks [83,88,89].

## Materials and Methods

### Animals and surgical procedures.

All experimental protocols were conducted according to the National Institutes of Health guidelines for animal research and were approved by the Institutional Animal Care and Use Committee at Cold Spring Harbor Laboratory. C57BL/6J mice (age postnatal day 18–25) were anesthetized using either ketamine (80 mg/kg) and xylazine (6 mg/kg), or urethane (1.5 g/kg), or isoflurane (0.25%–1% vol/vol O_2_) (Figures 1–4). The data in Figures 5–8 were collected under ketamine–xylazine anesthesia. During the imaging sessions, the level of anesthesia was monitored with LFP and/or electroencephalogram (EEG) recordings. The response probability remained constant during the experiments (Figure S2). Core body temperature was maintained at 37 °C using a heating blanket (Harvard Apparatus, http://www.harvardapparatus.com). Imaging windows were installed above the barrel cortex. A small craniotomy (diameter, 1–2 mm) was made 1 mm posterior from bregma and 3.5 mm lateral from the midline on the right hemisphere. The intact dura was covered with 2% agarose (Type-IIIA; Sigma, http://www.sigmaaldrich.com), dissolved in Hepes-buffered artificial cerebrospinal fluid, and a 5-mm cover glass (World Precision Instruments, http://www.wpiinc.com). The cover glass was then sealed in place using dental acrylic, leaving one side open [49,90]. To record the electrocorticogram (EcoG), a thin (0.2 mm) Teflon-coated silver wire was inserted between the dura and skull through a hole in the opposite hemisphere, and a reference wire was inserted above the cerebellum.

### Loading procedures.

Neocortical neurons were loaded with Fluo-4 AM (F14201; Invitrogen, http://invitrogen.com) using multicell bolus loading [22]. Fluo-4 AM was dissolved in 20% (w/v) Pluronic F-127 (P-6867; Invitrogen) in DMSO to a concentration of 10 mM. This solution was then diluted 10-fold into external buffer containing (in mM): 125 NaCl, 5 KCl, 10 Glucose, 10 Hepes, 2 CaCl_2_, 2 MgSO_4_, 0.05 Alexa 594. In some experiments, Alexa 594 was replaced with Sulforhodamine 101 (Invitrogen) to label astrocytes [45]. Glass micropipettes (tip resistance approximately 3 MΩ) were filled with the loading solution and inserted into the barrel cortex. Repetitive positive-pressure pulses (5 psi, 10 ms, 10–20 times; PicoSpritzer II; General Valve/Parker, http://www.parker.com) were applied to eject the dye to bulk load a small volume (diameter, approximately 200 μm) (Figure 1). The loading procedure did not change the response properties of the neurons, as assessed by the sensory-evoked LFP (LFP_after_/LFP_before_ = 103.4% ± 5.4%, *t*_6_ = 1.68, *n* = 7, *p* > 0.1).

### Two-photon microscopy.

In vivo imaging was performed using a custom-made two-photon laser-scanning microscope (TPLSM) controlled by ScanImage software [91]. The light source was a pulsed Ti:sapphire laser (λ ~ 810 nm; 50–150 mW in the objective back-focal plane; MaiTai; Spectra-Physics, http://www.spectraphysics.com). Red and green fluorescence photons were separated using a 565-nm dichroic mirror (Chroma Technology, http://www.chroma.com) and barrier filters (green, BG22; red, 607/45; Chroma). Signals were collected using photomultiplier tubes (3896; Hamamatsu Photonics, http://www.hamamatsu.com/). The objective lens (40, 0.8 NA) and trinoc were from Olympus (http://www.olympus.co.jp/en/). We used frame scanning (frame rate = 15.6 Hz) with 2-ms line scan durations (32 × 256 or 32 × 1,024 pixels). Pixel dimensions were 0.22 μm × 0.23 μm or 0.19 μm × 1.7 μm. Images were collected in L2/3, 120–250 μm from the top of the dura [25,29].

### Loose-seal cell-attached recording.

Cells were targeted for patching using the TPLSM fluorescence image [92]. The recording pipette contained (in mM): 10 KCl, 140 K-gluconate, 10 Hepes, 2 MgCl_2_, 2 CaCl_2_, 0.05 Alexa 594, and was adjusted to pH 7.25 and 290 mOsm. Loose-seal cell-attached recordings [92] were made from Fluo-4 AM–loaded L2/3 neurons. The signals were recorded using a patch-clamp amplifier (Axoclamp 200B; Axon Instruments, http://www.axon.com/), and APs were detected using thresholding [61]. At the end of the recording session, the recorded cells were filled with Alexa 594 by perforating the membrane with large-current injections. All data were collected using custom-written physiology software in MATLAB (The Mathworks, http://www.mathworks.com).

### Sensory stimulation.

The loading pipette was used to measure the sensory stimulation-evoked LFP. A hand-held stimulator was used to identify whiskers that were effective in evoking LFPs in the area loaded with Ca^2+^ indicator. These whiskers were then deflected using a piezoelectric stimulator (300-μm deflection in the rostral–caudal direction, positioned 5 mm from the base of the whisker) while recording the LFP and EcoG. The rise time (10%–90%) of the piezo movement was less than 2.8 ms. The fractional amplitude and the decay constant of the ringing were 48.6% and 49 ms, respectively. In a few experiments, the rise time was reduced to 8 ms, which abolished the ringing (fractional amplitude, 2.2%). The two stimuli produced indistinguishable responses in L2/3 neurons (unpublished data). The pair of whiskers evoking the largest LFP response (PW) and the second largest LFP response (SW) were used for further mapping.

Imaging sessions began 30 min after dye loading. Trial durations were either 3,200 ms (50 frames) (Figure 2A) or 768 ms (12 frames) (all figures other than Figure 2A). To minimize use-dependent depression, intertrial intervals were long (20–30 s) [93]. Whiskers were stimulated in an interleaved fashion. The imaging session lasted 1–1.5 h (200–300 trials). Two factors conspired against longer imaging sessions. First, Fluo-4AM is extruded from the neuronal cytoplasm. Second, extracellular baseline fluorescence increased with time due to unknown causes [94].

Following the imaging experiments, fluorescent beads were injected into the center of the imaging site. In most of the experiments, PW was verified anatomically by examining the barrel pattern in tangential sections (100-μm thick) through L4 stained with cytochrome oxidase [95,96] .

### Data analysis.

Single whisker deflections have been reported to induce zero or one, rarely two, APs in L2/3 neurons [12,26,27,32,49]. APs cause Ca^2+^ accumulations in somata and dendrites that can be detected with Ca^2+^ imaging in vivo [49,90,97]. Since individual dendrites were difficult to identify, we performed fluorescence measurements in somata. Somata were identified by combining the images from the green channel (Ca^2+^ dye) and the red channel (counterimage generated by Alexa 594 in the extracellular space) (Figure 1B). Regions of interest were manually selected inside the somata. We separated trials that resulted in AP-evoked fluorescence transients (successes) from trials that did not produce fluorescence transients (failures) using custom-written algorithms (see Results). The response probability (*P*_r_) was determined for each cell and whisker (PW and SW) as the number of responsive trials divided by the total number of trials. The SI was calculated for each cell as:

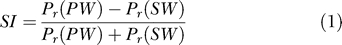



SI ranges from one (only responsive to PW) to minus one (only responsive to SW).

The SIs were compared across pairs of simultaneously imaged neurons using a bootstrap analysis. For each iteration of the bootstrap, the SI was calculated for each neuron by sampling with replacement from the lists of trials containing successes and failures. The resampling was performed separately for PW and SW stimuli, and then the differences between the SIs were calculated. For each pair of neurons, the distribution of the difference of the SI was generated. Ninety-five percent confidence intervals were used to determine whether the differences were significantly different from zero. The slopes of regression lines were compared using standard bootstrap analysis methods [98].

Trial-to-trial correlations were calculated for pairs of neurons imaged simultaneously. The number of trials in which both neurons fired was counted. This number was then divided by the number of trials in which the less-responsive neuron fired. This value represents the probability that the more-responsive neuron fires given that the less-responsive neuron fires.

To test for similarity between “raw data'” and “adjusted data” (Figure 8), the response matrix was randomized (1,000 times) by shuffling the trials for each neuron independently. For each shuffled response pattern, the correlation between “raw data” and “adjusted data” was calculated. The *p*-value was determined from the distribution of the correlations from shuffled data.

## Supporting Information

Figure S1 The Relationship between the Number of Responsive Neurons and the LFP Level at the Time of the Sensory Stimulus (A) The LFP level was calculated as the difference between the local maximum and the LFP at the time of sensory stimulation (averaged over the times from −10 ms to 0 ms). The LFP level is highly correlated with UP and DOWN states [99,100]. Negative LFP levels correspond to UP states, as assessed by increased multiunit activity (unpublished data). Each circle represents one trial (regression line, red).
(B) The distribution of the correlation coefficient between the LFP level and the number of responsive neurons across 21 experiments.
(101 KB PDF)

Figure S2 The Response Probability to PW Stimulation over Time (Averaged over All Experiments) (193 KB PDF)

Figure S3 The Difference in the SI for Pairs of Neurons as a Function of the Distance between the Neurons In contrast to Figure 7D, here we used the absolute distance.
(275 KB PDF)

Video S1 Spontaneous Activity in the Mouse Barrel Cortex The sound corresponds to an AP recorded in the neuron located close to the center of the image. The movie shows raw data without background subtraction.
(7.8 MB AVI)

## Abbreviations

AP: action potential
L: layer
LFP: local field potential
PW: principal whisker
SI: selectivity index
SW: surround whisker
