# Supplementary figures and images for: The Functional Microarchitecture of the Mouse Barrel Cortex
Source: PLoS Biol. 2007 Jul 10;5(7):e189. doi: 10.1371/journal.pbio.0050189 (PMC1914403; doi:10.1371/journal.pbio.0050189)

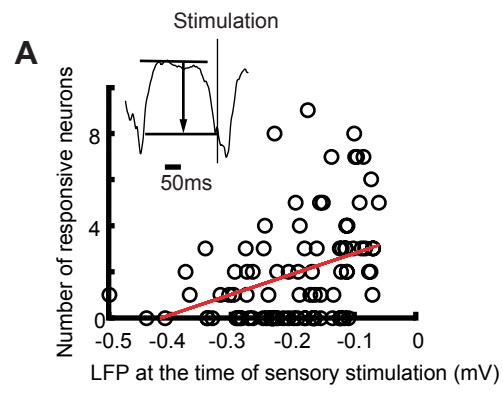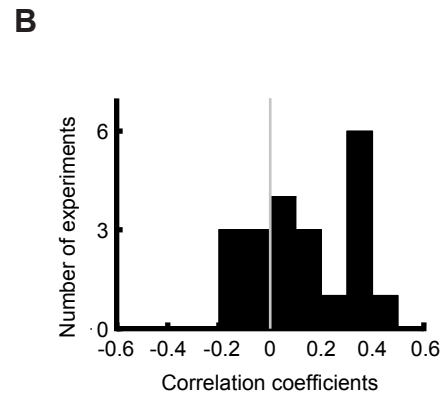

Supplementary Figure 1

Supplement: Figure S1 — (A) The LFP level was calculated as the difference between the local maximum and the LFP at the time of sensory stimulation (averaged over the times from −10 ms to 0 ms). The LFP level is highly correlated with UP and DOWN states [99,100]. Negative LFP levels correspond to UP states, as assessed by increased multiunit activity (unpublished data). Each circle represents one trial (regression line, red). (B) The distribution of the correlation coefficient between the LFP level and the number of responsive neurons across 21 experiments. (101 KB PDF) [file pbio.0050189.sg001.pdf]

Stability of sensory response over trials

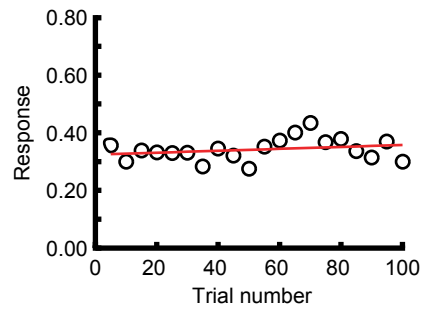

Supplementary Figure 2

Supplement: Figure S2 — (193 KB PDF) [file pbio.0050189.sg002.pdf]

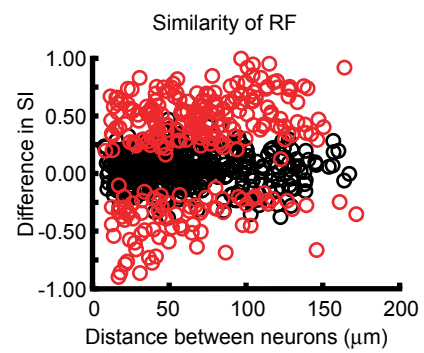

Supplementary Figure 3

Supplement: Figure S3 — In contrast to Figure 7D, here we used the absolute distance. (275 KB PDF) [file pbio.0050189.sg003.pdf]
